# Is living on the coast associated with midlife multimorbidity in England and Wales? A cross-birth cohort analysis of the relationship between long-term residence and selective migration

**DOI:** 10.1136/bmjph-2025-004020

**Published:** 2026-07-30

**Authors:** Stephen Jivraj, Zongpu Yue, Avril Keating, Emily Murray

**Affiliations:** 1 Institute of Epidemiology and Health Care, UCL, London, UK; 2 Institute of Education, UCL, London, UK; 3 Institute of Public Health and Wellbeing, University of Essex, Colchester, UK

**Keywords:** Epidemiologic Factors, Comorbidity, Demography, Public Health, Sociodemographic Factors

## Abstract

**Introduction:**

The 2021 Chief Medical Officer’s report highlighted that coastal communities have some of the worst health outcomes in England. The drivers of this spatial disadvantage are unclear. Moreover, some evidence suggests that living closer to the coast has protective health effects. This paper investigates the relationship between coastal residence and multimorbidity and assesses the role of selective migration and socioeconomic deprivation from adolescence to midlife in shaping health outcomes in coastal communities.

**Methods:**

Longitudinal cohort study data are used from the 1958 National Child Development Study (NCDS) and the 1970 British Cohort Study (BCS). Coastal residence is defined using the Office for National Statistics’ built-up areas in England and Wales. The analysis includes 13 691 respondents from NCDS and 14 683 from BCS, followed from birth through adolescence and midlife. Multimorbidity in midlife, defined as the presence of two or more doctor-diagnosed chronic conditions at age 55 years (NCDS) and age 46 years (BCS), is the outcome in the analysis.

**Results:**

Although no association was found between coastal residence in adolescence and multimorbidity in midlife, coastal residence in midlife was associated with 1.21 (95% CI 1.00 to 1.28) higher odds of multimorbidity later in life in the earlier-born cohort, though this was attenuated by neighbourhood deprivation at age 42 years. Individuals who moved away from coastal areas between adolescence and midlife were significantly (p<0.05) less likely to report multimorbidity than those who moved to coastal areas. Neighbourhood deprivation was strongly associated with migration patterns, with those remaining in or moving to coastal areas significantly (p<0.05) more likely to live in deprived neighbourhoods at midlife.

**Conclusions:**

Coastal communities in England and Wales are experiencing a concentration of health disadvantage that is associated with socioeconomic deprivation and patterns of selective migration. These findings have important implications for public health policy and resource allocation in coastal regions.

**WHAT IS ALREADY KNOWN ON THIS TOPIC**

- Coastal living in Britain has been associated with both health benefits and disadvantages.
- Previous studies have relied on cross-sectional or ecological data, limiting causal inference.

**WHAT THIS STUDY ADDS**

- Living in a coastal area in adolescence is not associated with multimorbidity in midlife among those born in the 1958 or 1970 cohorts.
- Coastal residence in midlife is linked to a higher probability of multimorbidity later in life, but only in the earlier-born cohort and before adjusting for neighbourhood deprivation.
- Individuals who move away from coastal areas are significantly less likely to report multimorbidity than those who move to or remain in coastal areas.
- Coastal areas are increasingly characterised by socioeconomic deprivation and a concentration, through selective migration, of health disadvantage.

**HOW THIS STUDY MIGHT AFFECT RESEARCH, PRACTICE OR POLICY**

- Public health strategies should be aware of the role of selective migration and deprivation in shaping health outcomes in coastal communities.
- Coastal areas may require targeted health interventions and resource allocation to address growing multimorbidity.

## Introduction

 There are two competing perspectives on the relationship between living on the coast and health in contemporary Britain. The first perspective suggests that living close to the coast is beneficial for health.^1–4^ The benefits of coastal living are found for subjective mental distress^1,2^ and self-reported general health.^2–4^ The reasons given for this perspective are fourfold: the setting of a coastal environment can refocus attention away from the stresses of everyday life; coastal environments can support healthy behaviours, such as walking; coastal environments promote social interactions and there is less pollution in coastal environments.^5,6^ The idea that the coast and its aesthetic quality can provide an equigenetic protective effect against poor health has a long tradition. ‘Equigenesis’ is a term that comes out of the health field to refer to when something in the environment disrupts the usual relationship between economic disadvantage and a poor health outcome, making lower and higher economic status groups more equal.^7^ Many of the seaside towns in Britain were founded as health resorts on the recommendation of early orthodox medicine’s advocacy of the therapeutic effects of salt air, salt water and sea bathing as a treatment for disease.^8,9^

An alternative perspective is that living on the coast is associated with poorer health. Asthana and Gibson^10^—drawing on their analysis that was used in the 2021 Chief Medical Officer’s annual report on health in coastal communities—found a higher prevalence of non-communicable diseases around the coastal fringe of England using pooled general practice data (2014–2019). In particular, coronary heart disease prevalence is at least 0.5 percentage points higher in coastal areas compared with non-coastal areas.^10^ The authors suggest that this is a consequence of the older age profile of coastal communities. The Office for National Statistics’^11^ profile of coastal communities in England and Wales using 2021 Census data suggests that inequality in health is present in age-standardised estimates of good health. They find that the percentage of the population in coastal communities reporting good health is two percentage points lower compared with non-coastal communities. There is also a suggestion that growing up in the most deprived coastal communities in England around the turn of the 21st century was associated with a greater likelihood of early-adulthood long-standing illness or disability and mental distress.^12^

The vices of many coastal areas are attributed to the decline in their traditional industries and a failure to develop alternative employment opportunities beyond tourism.^10^ The uncertainty of domestic tourism has resulted in acute economic disadvantage in selected coastal areas, which has led to a plethora of broader social consequences.^13–15^ Although economic decline is not unique to coastal areas, it has been argued that social disadvantage in coastal areas is harder to overcome relative to inland places because of the lack of connectivity to centres of employment and education, as a consequence of being at the end of the line, with the sea acting as a natural barrier that is not faced by inland communities.^15,16^ Coastal areas are also at the frontline of environmental threats brought about by climate change and water pollution that could affect the health of the people resident in these places.^13^

A potential explanation for the difference in findings could be different definitions of how living on the coast is operationalised. The literature suggesting a protective effect of the coastal proximity in Britain exclusively defines coastal living by distance to the coast in categories of thresholds (eg, 0–1 km; >1–5 km; >5–20 km; >20–50 km; >50 km) using population-weighted centroids of lower super output areas (LSOAs), a census output geography widely used to define neighbourhoods. It could be the case that measuring coastal living by categories of distance is a proxy for access to the coast and may underestimate the stark difference of living on the coast or not. There is theoretical reasoning for this in that the benefits of the coast are often realised when someone is in a leisure state of mind, and, therefore, those living on the coast may not have their mental receptors as open to its positive impact.^17^

By contrast, research suggestive of a deleterious effect of coastal living in Britain exclusively uses binary definitions, using either LSOAs within 500 m of the coast or built-up areas (BUAs). BUAs are derived by the Office for National Statistics using Ordnance Survey topographic data to define the perimeter of settlements and identify which cities, towns and villages are coastal.^11^ Coastal BUAs are defined by distance of a boundary within 1 km of the coastline and most of their surface area within 3 km of the coastline, or they have 2.5 km of their boundary within 25 m of the coastline. This definition of coastal residence is used in this paper as a refined measure that is unlikely to misclassify a locality as coastal. Misclassification is more likely using LSOAs because they do not encompass settlements and could result in people living on one side of a street living in a coastal area and those on the other side of the street not living in a coastal area.

Broader limitations of much of the existing literature on the relationship between coastal living and health include a reliance on ecological or cross-sectional data, which limits the ability to draw inferences about individuals and to demonstrate causality. Exceptions are White *et al*’s^2^ study using the British Household Panel Study and Murray *et al*’s^12^ analysis of the UK Household Longitudinal Study. Both studies have limitations that this current study aims to build on, including a limited or unclear period of follow-up. The current study analyses the relationship between coastal living from adolescence up to age 42 years and health at either age 55 years using the 1958 National Child Development Study (NCDS) or age 46 years using the 1970 British Cohort Study (BCS).

The use of individual repeated-measures (ie, longitudinal) data to test diagnosed health outcomes is absent from the existing literature on the relationship between coastal living and health in the UK and beyond. The outcome used in the current study is multimorbidity. Multimorbidity is defined as the presence of two or more doctor-diagnosed chronic conditions, representing an increasingly important challenge to healthcare systems and broader health-related policymaking.^18–20^ The implications of the analysis of multimorbidity will provide evidence directly related to the demand for healthcare services.

Little is known about whether the relationship between coastal living and health changes over the life course or whether it has changed over different periods. It could be the case that the composition of coastal communities has changed in recent times because of selective migration due to either or both sicker people moving in and less sick people moving out. It could also be the case that those left behind in coastal areas are at a greater predisposition to poor health compared with people who move to and from coastal areas, as well as those who remain inland. The changing socioeconomic and built environment of the coast might also bring about worse contemporary health outcomes in a way that it did not, relative to inland areas, in the past.

The current paper provides answers to the following research questions:

1. Is growing up in a coastal area associated with multimorbidity in midlife?
2. Is living in a coastal area in midlife associated with multimorbidity later in life?
3. Does selective migration explain any differences in the answers to questions (1) and (2)?
4. How is neighbourhood deprivation associated with immobility and migration into and out of coastal areas from childhood to midlife?

## Methods

### Data

The 1958 NCDS and the 1970 BCS are described as the crown jewels of British social science.^21^ The two studies follow the lives of around 17 000 people from birth in a single week of 1958 or 1970 who were living in England, Wales or Scotland. The original respondents have been followed up over the course of their lives up to the age of 62 years in NCDS and 51 years in BCS where information has been collected regarding their health, social development and economic circumstances. Data are used here for those living in England or Wales from birth up to midlife: age 55 years in NCDS and age 46 years in BCS.

### Measures

#### Outcome variable

Multimorbidity is measured from a count of 9 health conditions (physical and mental) that respondents report having had in the last 4–5 years, whether newly reported or recurring, at the age of 55 years in NCDS and 46 years in BCS. The conditions are asthma, diabetes, backache, cancer, hearing problems, high blood pressure, eye disease, heart problems and depression. A count of two or more of these conditions indicates multimorbidity. Respondents with missing values on two or more conditions, who reported two or more missing values across the conditions, were set to missing, as were respondents with one condition but missing values on at least one other.

#### Exposure variables

**Figure 1:**
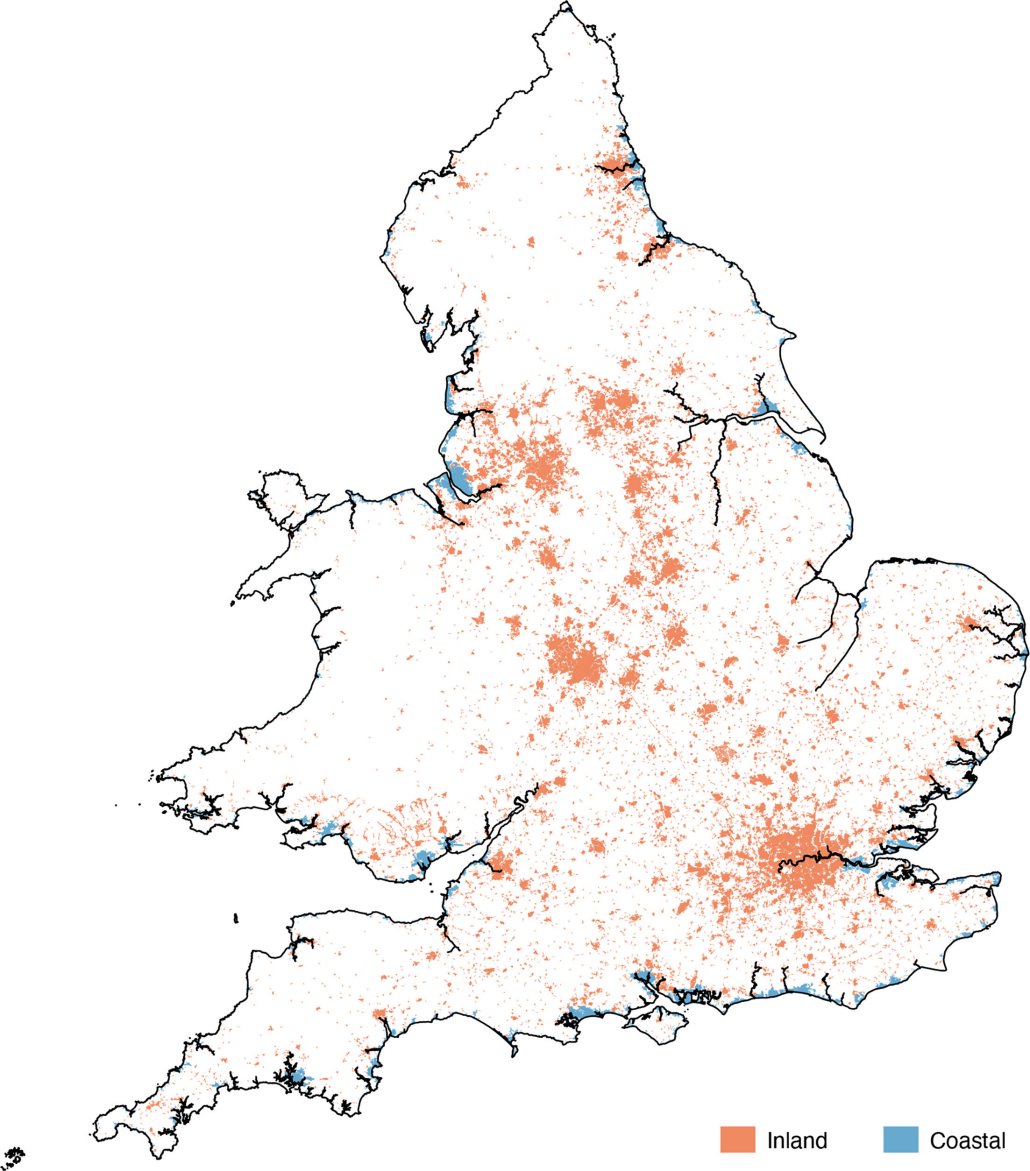
Built-up areas in England and Wales by coastal status.

Coastal areas are identified by linking an NCDS or BCS respondent’s postcode at ages 16 years and 42 years to the National Statistics Postcode Directory (NSPD). The NSPD contains identifiers for BUAs, which have been classified by the Office for National Statistics according to the description in the previous section. Figure 1 shows the identification of coastal and inland BUAs. There are a small number of respondents who do not live in a BUA (5% in NCDS and 7% in BCS at age 16 years). These include people living on farms and in other remote locations, and have been classified as non-coastal.

Migration between coastal and non-coastal areas or vice versa is measured by the change in coastal residence between ages 16 and 42 years. Four categories of migration status are used: inland, coastal, moved to the coast and moved away from the coast.

#### Control variables

To take account of the socioeconomic context of coastal areas, which are generally more deprived,^10^ neighbourhood deprivation deciles were linked to respondents at ages 16 years and 42 years. Neighbourhood deprivation is measured by deciles on the national distribution of Townsend index values using data from the 1971 to 2011 census data, interpolated and reapportioned to LSOA boundaries in 2011.^22,23^ Individual socioeconomic position is included in the analysis to take account of selection into neighbourhoods^24,25^ and a pathway to multimorbidity^26,27^ throughout the life course and is measured using social class at the birth of a respondent’s father using the Registrar General’s occupational classification.^28^ The classes are professional occupations (eg, doctors and lawyers), managerial and technical occupations (eg, teachers and nurses), skilled non-manual occupations (eg, clerical workers and secretaries), skilled manual occupations (eg, electricians and plumbers), partly skilled occupations (eg, postal workers and bar staff) and unskilled occupations (eg, labourers and cleaners). Approximately 5% of respondents in NCDS and 7% of respondents in BCS were not classifiable. Gender at birth is also included in the analysis, given the different prevalence of multimorbidity between men and women.

### Statistical analysis

Separate logistic regression models were fitted to predict multimorbidity at age 55 years (in NCDS) and 46 years (in BCS) without adjustment for control variables, and then with adjustment for control variables. To answer research question (1), coastal living status at age 16 years was added to the model; to answer question (2), coastal living status at age 42 years was added to the model. A further stage of adjustment, controlling for multimorbidity status at age 42 years, is added to the model to see whether coastal living from midlife is predictive of multimorbidity at a slightly later age. Heart problems were not recorded at age 42 years in BCS, and, therefore, the multimorbidity count includes one fewer condition at this age.

To answer question (3), a separate model was fitted using multimorbidity at either age 55 years (NCDS) or 46 years (BCS) as the outcome and migration status between ages 16 and 42 years as an alternative exposure to coastal living status at age 16 years. This model contains all control variables, including multimorbidity status at age 42 years. Question (4) was answered using multinomial logistic models fitted with migration status as the outcome and neighbourhood deprivation at ages 16 years and 42 years as the only explanatory variables.

To take account of missing data at each wave and longitudinally from birth to age 46 years in BCS and 55 years in NCDS, multiple imputation by chained equations was used to deal with bias in non-response. The imputation models include auxiliary variables shown to be associated with missingness in order to bring the sample size to its maximum total, minus those known to have died or emigrated. A separate imputation model is fitted for both cohorts, and within each cohort, one imputation model is fitted to answer questions 1–2 and a separate imputation model is fitted for question 3. The imputation models contain all variables included in each model of interest, as well as more than 20 auxiliary variables listed in the Centre for Longitudinal Studies’ missing data strategy.^29^ Truncated regression was used in the imputation models when they allowed convergence. For example, imputed values for the neighbourhood deprivation index were constrained to lie between 1 (least deprived) and 10 (most deprived). The number of imputations in each model was 25, given that coefficients and SEs did not substantially change compared with a smaller number of imputed datasets in each model of interest. The sample size used in the NCDS analysis was 13 691 and 14 683 in the BCS analysis, which would be 5391 in NCDS and 5161 in BCS using a complete case sample. Regression estimates are shown in the online supplemental appendix with and without imputed data. The broad substantive findings are not different whether using a complete case analysis or imputed data. See table 1 for sample characteristics using observed data from birth, age 16 years, age 42 years and either age 46 years in BCS or age 55 years in NCDS.

**Table 1:** Sample characteristics

	NCDS		BCS	
	Mean (SD) or percentage	Percentage missing (%)	Mean (SD) or percentage	Percentage missing (%)
Multimorbidity				
Age 55 years (%)	27.78	42.05		
Age 46 years (%)			19.77	47.61
Age 42 years* (%)	10.58	29.77	14.40	40.51
Coastal residence				
Age 16 years (%)	15.58	26.63	15.72	34.25
Age 42 years (%)	15.24	29.62	15.71	40.50
Migration status age 16–42 years				
Inland at both ages (%)	78.70	43.38	78.24	54.21
Coastal at both ages (%)	9.79		9.80	
Moved to coast (%)	5.22		5.77	
Moved inland (%)	6.28		6.19	
Neighbourhood deprivation				
Age 16 years	5.94 (2.75)	26.64	5.35 (2.88)	34.26
Age 42 years	4.82 (2.75)	29.62	4.85 (2.69)	40.52
Sex				
Males (%)	50.84	0.00	51.14	0.01
Social class at birth				
I (%)	4.58	11.42	5.02	8.24
II (%)	13.33		13.09	
III (non-manual) (%)	10.04		13.69	
III (manual) (%)	50.45		44.87	
IV (%)	12.11		16.48	
V (%)	9.50		6.84	

* Multimorbidity does not contain heart problems in BCS at age 42 years.

BCS, 1970 British Cohort Study; NCDS, 1958 National Child Development Study.

### Patient and public involvement

Patients and the public were not involved in the design or implementation of the study or in the interpretation or writing up of results. Dissemination of findings to study participants will be conducted as part of the broader dissemination activity of the Centre for Longitudinal Studies (https://cls.ucl.ac.uk/).

## Results

**Figure 2:**
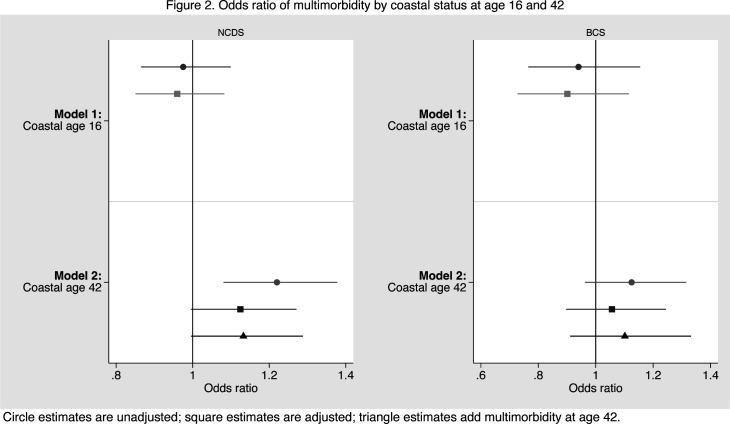
OR of multimorbidity by coastal status at ages 16 years and 42 years. BCS, 1970 British Cohort Study; NCDS, 1958 National Child Development Study.

Figure 2 shows the OR of multimorbidity for NCDS and BCS respondents living in a coastal BUA at ages 16 years and 42 years compared with those not living in a coastal BUA. The circles show the unadjusted estimates, and the squares show the adjusted estimates. Living in a coastal BUA at age 16 years was not associated with higher or lower odds of multimorbidity at age 55 years in the NCDS sample, irrespective of level of adjustment. Also in the unadjusted analysis, living in a coastal BUA at age 42 years was associated with a 22% (OR 1.22 (95% CI 1.08 to 1.38)) higher odds of multimorbidity at age 55 years compared with living in a non-coastal BUA. The latter difference between coastal and non-coastal BUAs is not significant after adjustment for neighbourhood deprivation at age 42 years and, as shown by the triangle estimates, was not sensitive to adjustment for multimorbidity at age 42 years. The findings in the BCS sample are similar. In the BCS sample, there was no association between living in a coastal BUA at age 16 years and multimorbidity at age 46 years. In contrast to the earlier-born cohort, there was no significant difference in the odds of multimorbidity at age 46 years in the BCS sample by coastal residence at age 42 years.

**Figure 3:**
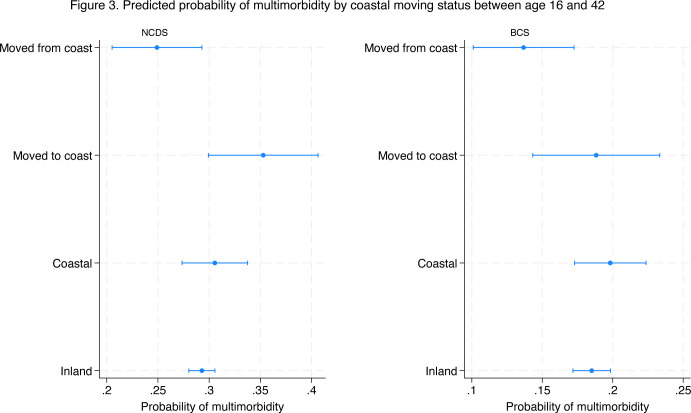
Predicted probability of multimorbidity by coastal moving status between ages 16 and 42 years. BCS, 1970 British Cohort Study; NCDS, 1958 National Child Development Study.

Figure 3 shows predicted probabilities of multimorbidity at age 55 years in NCDS and 46 years in BCS by migration status between ages 16 and 42 years at the mean value of gender and social class at birth, neighbourhood deprivation at age 16 years and multimorbidity status at age 42 years. Those moving to the coast between these ages had the highest probability of multimorbidity in the NCDS sample (35% (95% CI 29.92% to 40.65%)), followed by those living on the coast at both ages (31% (95% CI 27.30% to 33.81%)), those living inland at both ages (29% (95% CI 28.00% to 30.58%)) and those moving away from the coast (25% (95% CI 20.51% to 29.30%)). The difference between those living either on the coast or inland at both time points is not significant. The difference between the two moving groups was significant, as was the difference between those moving from the coast, and those remaining on the coast and the difference between those moving to the coast and those remaining inland. Similar patterns across migration statuses in the probability of multimorbidity at age 46 years are shown in the BCS sample. Those living on the coast at both ages had the highest probability of multimorbidity (19.83% (95% CI 17.24% to 22.42%)), followed by those moving to the coast (18.83% (95% 14.31% to 23.34%)), those living inland at both ages (18.51% (95% CI 17.15% to 19.87%)) and those moving away from the coast (13.68% (95% CI 10.10% to 17.25%)). There was no significant difference between moving to the coast and those who either stay on the coast or stay inland. In contrast, the lower probability of multimorbidity in those moving away from the coast was significantly different from all other migration statuses.

Table 2 shows the relative risk ratios of (1) remaining in a coastal area, (2) moving to a coastal area and (3) moving from a coastal area compared with the base outcome of remaining in an inland area in NCDS and BCS. In the NCDS sample, the relative risk of remaining in a coastal area to remaining in an inland area increases by a factor of 1.05 for each decile increase in neighbourhood deprivation at age 16 years and at age 42 years by a factor of 1.06. This means that respondents living in coastal areas compared with inland areas at either age 16 years or 42 years typically lived in more deprived neighbourhoods. The same was the case in the BCS sample, where there was a slightly stronger association.

**Table 2:** Relative risk ratios for moving status between ages 16 and 42 years (base outcome in multinomial model is remaining in an inland BUA)

	NCDS				BCS			
	RRR	P value	95% CI		RRR	P value	95% CI	
Remaining in coastal BUA								
Neighbourhood deprivation age 16 years	1.036	0.009	1.010	1.064	1.090	0.000	1.060	1.121
Neighbourhood deprivation age 42 years	1.094	0.000	1.066	1.122	1.071	0.000	1.045	1.101
Move to a coastal BUA								
Neighbourhood deprivation age 16 years	0.925	0.000	0.891	0.960	0.932	0.001	0.895	0.970
Neighbourhood deprivation age 42 years	1.038	0.074	0.996	1.080	1.046	0.041	1.002	1.092
Moving from a coastal BUA								
Neighbourhood deprivation age 16 years	1.008	0.649	0.973	1.043	1.036	0.073	0.997	1.077
Neighbourhood deprivation age 42 years	0.867	0.000	0.834	0.901	0.857	0.000	0.820	0.895

BCS, 1970 British Cohort Study; BUA, built-up area; NCDS, 1958 National Child Development Study; RRR, Relative Risk Ratios.

For each unit increase in neighbourhood deprivation decile at age 16 years, respondents were less likely to move to a coastal area between the ages of 16 years and 42 years compared with remaining in an inland area by a factor of 0.93 (95% CI 0.89 to 0.96) in NCDS and 0.93 (95% CI 0.89 to 0.97) in BCS. This suggests that respondents were less likely to move to coastal areas from less deprived inland neighbourhoods than from more deprived inland areas. There was no significant difference by neighbourhood deprivation at age 42 years in the odds of moving to a coastal area in the NCDS sample. However, the relative risk ratio of moving to a coastal area to remaining in an inland area was 1.05 (95% CI 1.00 to 1.09) for each increase in neighbourhood deprivation at age 42 years in the BCS sample. This means people moving to coastal areas were more likely to move to more deprived coastal neighbourhoods compared with less deprived coastal neighbourhoods.

The odds of moving away from a coastal area to staying in an inland area by neighbourhood deprivation decile at age 16 years were not significant in the NCDS or BCS samples. However, a unit increase in neighbourhood deprivation at age 42 years multiplies the odds of moving away from a coastal area compared with remaining in an inland area by 0.87 (95% CI 0.83 to 0.90) in NCDS and 0.85 (95% CI 0.82 to 0.90) in BCS. These results suggest that respondents were moving from more to less deprived neighbourhoods when leaving coastal areas between the ages of 16 and 42 years.

## Discussion

Multimorbidity presents an increasingly important global public health challenge tied to ageing populations and increased socioeconomic inequality.^18^ This paper is the first in England and Wales to compare multimorbidity between those living in coastal areas and inland areas and by taking a life-course perspective from childhood to midlife.

The paper’s first objective sets out to determine whether growing up in a coastal area in England and Wales is associated with multimorbidity later in life. We find no evidence that those born in the third quarter of the 20th century were any more or less likely to report having two or more chronic conditions (ie, multimorbidity) in midlife if they grew up in a coastal area compared with an inland area. Simply growing up in a coastal community does not, in and of itself, predict a greater likelihood of multimorbidity. This supports existing evidence showing that growing up in deprived areas more generally is not predictive of adverse health outcomes in midlife using the same data as this study.^30^

Our second objective was to determine whether living in a coastal area in midlife was associated with multimorbidity at an age slightly later in life. There is an unadjusted association between living in a coastal area by the age of 42 years and a higher probability of multimorbidity at age 55 years in the NCDS sample (born 1958) compared with living inland. The elevated odds of more than 20% are largely explained by neighbourhood deprivation at age 42 years. The findings are the same for the incidence of multimorbidity between ages 42 and 46 years in both cohorts.

Our third objective was to establish whether selective migration explains part of the relationship between health and coastal living in midlife. We find compelling evidence to suggest that those moving out of coastal areas between the ages of 16 and 42 years are much less likely to report multimorbidity at midlife compared with those who move into coastal areas between the same ages. The absolute difference in the predicted probability of multimorbidity between in-migrants and out-migrants is 10 percentage points in NCDS at age 55 years and 5 percentage points in BCS at age 46 years. This means that selective migration makes the composition of coastal areas unhealthier. The nature of this selective migration process between coastal and inland areas is not restricted to the 1958 and 1970 birth cohorts. Graduates entering the labour market in the 2010s who left coastal areas to attend university have been shown to be less likely to return to reside in coastal areas after graduation compared with those who left to attend university from inland areas in England.^31^ This builds on a general perception that many young people who are able to leave coastal areas do so.^32^ What is not clear is whether people moved away from coastal areas because the area declined economically or whether these places declined because people moved away.

Our analysis of the association between neighbourhood deprivation at ages 16 years and 42 years and moving between coastal and inland areas, or remaining in one or the other, suggests that those remaining in coastal areas lived in more deprived neighbourhoods in adolescence and in midlife compared with those who remained living inland. Moreover, those who moved to coastal areas were more likely to move from less deprived areas and to more deprived neighbourhoods, and those who moved from coastal areas were more likely to move from more deprived areas and to less deprived areas compared with those who remained inland. This suggests that the population is more concentrated in deprived areas when living in coastal areas, regardless of migration, and that the process of selective migration is increasing this concentration. Given the relationship with health and migration, deprived coastal areas are becoming more concentrated with multimorbid populations over time. This trend of movement of people in midlife amplifying health inequalities by neighbourhood deprivation has been found generally in England and Wales.^33^

The findings of this paper, while they do not definitively support a causal effect of coastal living on health, have important public health implications. The evidence presented here suggests that population health in coastal areas is becoming worse, especially in coastal towns that are deprived. This is a result of people who grew up and stayed in coastal areas becoming more likely to develop morbidity from midlife compared with those staying in inland areas and the balance of selective migration concentrating multimorbidity in coastal areas: the people who move to coastal areas are sicker compared with those who move away from coastal areas. An intergenerational consequence of these processes may explain why young people growing up in coastal areas in the early part of the 21st century report worse mental health compared with their inland counterparts.^12^ It may also contribute to the reciprocal spiral of decline in some coastal areas which have experienced considerable downturns in their economic capacity, which in turn limit productive opportunities for inhabitants and cause worse population health. Further research could explore the causal effects of moving to and from coastal areas to determine whether those who move into coastal areas would have fared worse in terms of later-life health had they remained inland.

There are several limitations that the current paper has not been able to fully overcome. The first is the definition of what constitutes a coastal area. While using BUAs is arguably the most considered and comprehensive identification of coastal settlements used in the literature, there is potential for misclassification. For example, Middlesbrough on the Tees Estuary is not classified as coastal, whereas Southend on the Thames Estuary is coastal. The treatment of large coastal cities is also a problem because large coastal BUAs, such as Liverpool, contain areas, for example, Croxteth, which are further away from the coastline compared with separate inland BUAs. The second limitation is the crude categorisation of all coastal areas as one entity. Coastal BUAs in England and Wales spread the length and breadth of the country and comprise settlements with contrasting local economies, from fishing ports to entertainment centres and quaint holiday villages to industrial city ports.^32^ Further research could consider typologies of coastal areas and inland areas using larger samples than are available from NCDS and BCS.

A third limitation is the categorisation of migration status. This paper uses transitions between ages 16 and 42 years, which may misclassify some people who were transitory in a coastal area or an inland area at either or both ages. Future work could aim to develop fuller accounts of migration histories to determine the length of time spent living in a coastal area over the life course. The identification of living in a coastal area at age 16 years as a proxy for whether someone grew up in a coastal area is a further limitation. Both this and the previous limitation were unavoidable, given that the first age at which residential information is available in NCDS was 16 years and that the limited number of follow-ups across the life course means it is difficult to derive a complete residential history.

A fourth limitation is the differing age at which the outcome is measured in the NCDS and BCS samples, which limits cross-cohort comparison. This limitation is compounded by the fact that multimorbidity was measured with a subtle difference in the BCS sample at age 42 years, when heart problems were not recorded. These limitations could be overcome in further research by using more recent data at the same age, when available, and using the same set of conditions. An insurmountable limitation with the data used in this study is the fact that chronic conditions were self-reported. Evidence suggests that survey respondents tend to under-report chronic conditions, and there is some suggestion that this is more prevalent among lower socioeconomic status groups.^34,35^ This points to a potential underestimation of the multimorbidity burden in coastal areas, given that they are typically more deprived.

In conclusion, this paper suggests that selective migration between coastal and inland areas between childhood and midlife is an important determinant of the higher rate of multimorbidity in coastal areas compared with inland areas. This, coupled with the higher concentrations of deprivation in coastal areas, should be considered in the identification of public health needs.

## Supplementary material

10.1136/bmjph-2025-004020 online supplemental file 1

## Data Availability

Data may be obtained from a third party and are not publicly available.
