# Supplementary material for: Is living on the coast associated with midlife multimorbidity in England and Wales? A cross-birth cohort analysis of the relationship between long-term residence and selective migration
Source: BMJ Public Health. 2026 Jul 30;4(3):e004020. doi: 10.1136/bmjph-2025-004020 (PMC13436097; doi:10.1136/bmjph-2025-004020)
Supplement: Supplementary file 1 [file bmjph-4-3-s001.docx]

Regression results using imputed data

Multimorbidity at age 55 (NCDS)/ age 46 (BCS) and coastal living at age 16

|  | NCDS |  | BCS |  |
| --- | --- | --- | --- | --- |
|  | Unadjusted | Adjusted | Unadjusted | Adjusted |
| Coastal age 16 | 0.975 | 0.96 | 0.94 | 0.901 |
| Neighbourhood deprivation age 16 |  | 1.058** |  | 1.043** |
| Female |  | 1.014 |  | 1.235** |
| Social class (ref: V) |  |  |  |  |
| VI |  | 0.705** |  | 0.791 |
| III manual |  | 0.795** |  | 0.855 |
| III non-manual |  | 0.846 |  | 0.675* |
| II |  | 1.124 |  | 0.725* |
| I |  | 0.965 |  | 0.589** |
| Intercept | 0.436** | 0.325** | 0.269** | 0.241** |
| Number of observations | 13691 | 13691 | 14683 | 14683 |
| ** p<.01, * p<.05 |  |  |  |  |

Multimorbidity at age 55 (NCDS) and coastal living at age 42

| NCDS | Unadjusted | Adjusted | Prior MM adjustment |
| --- | --- | --- | --- |
|  |  |  |  |
| Coastal age 42 | 1.22** | 1.125 | 1.133 |
| Neighbourhood deprivation age 42 |  | 1.102** | 1.087** |
| Female |  | 1.017 | 1.066 |
| Social class (ref: V) |  |  |  |
| VI |  | 0.68** | 0.705** |
| III manual |  | 0.78** | 0.768** |
| III non-manual |  | 0.852 | 0.889 |
| II |  | 1.124 | 1.096 |
| I |  | 0.957 | 0.9 |
| Multimorbidity age 42 |  |  | 5.619** |
| Intercept | 0.421** | 0.268** | 0.225** |
| Number of observations | 13691 | 13691 | 13691 |
| ** p<.01, * p<.05 |  |  |  |

Multimorbidity at age 46 (BCS) and coastal living at age 42

| BCS | Unadjusted | Adjusted | Prior MM adjustment |
| --- | --- | --- | --- |
|  |  |  |  |
| Coastal age 42 | 1.125 | 1.057 | 1.102 |
| Neighbourhood deprivation age 42 |  | 1.075 ** | 1.061 ** |
| Female |  | 1.250 ** | 1.151 * |
| Social class (ref: I) |  |  |  |
| II |  | 0.808 | 0.882 |
| III non-manual |  | 0.876 | 0.908 |
| III manual |  | 0.684 * | 0.744 |
| IV |  | 0.735 * | 0.781 |
| V |  | 0.587 ** | 0.673 |
| Multimorbidity age 42 |  |  | 9.168 ** |
| Intercept | 0.262 ** | 0.200 ** | 0.129 ** |
| Number of observations | 14683 | 14683 | 14683 |

Multimorbidity at age 55 (NCDS)/ age 46 (BCS) and moving status between coastal and inland areas at age 16 and 42

|  | NCDS | BCS |
| --- | --- | --- |
|  |  |  |
| Moving status (ref: remain inland) |  |  |
| Remain coastal | 1.062 | 1.088 |
| Move to coast | 1.316* | 1.021 |
| Move from coast | 0.8 | 0.697 * |
| Neighbourhood deprivation age 16 | 1.05** | 1.038 ** |
| Female | 1.078 | 1.139 |
| Social class (ref: I) |  |  |
| II | 0.73* | 0.853 |
| III non-manual | 0.786** | 0.884 |
| III manual | 0.874 | 0.727 * |
| IV | 1.114 | 0.765 |
| V | 0.916 | 0.674 * |
| Multimorbidity age 42 | 5.761** | 9.085 ** |
| Intercept | 0.259** | 0.150 ** |
| Number of observations | 13691 | 14683 |

**Regression results using complete case data**

Multimorbidity at age 55 (NCDS)/ age 46 (BCS) and coastal living at age 16

|  | NCDS |  | BCS |  |
| --- | --- | --- | --- | --- |
|  | Unadjusted | Adjusted | Unadjusted | Adjusted |
| Coastal age 16 | 0.91 | 0.861 | 0.938 | 0.929 |
| Neighbourhood deprivation age 16 |  | 1.046 ** |  | 1.044** |
| Female |  | 1.174 * |  | 0.965 |
| Social class (ref: V) |  |  |  |  |
| VI |  | 0.731 |  | 0.803 |
| III manual |  | 0.783 |  | 0.796* |
| III non-manual |  | 0.637 ** |  | 0.926 |
| II |  | 0.739 |  | 1.153 |
| I |  | 0.576 ** |  | 0.888 |
| Intercept | 0.247 ** | 0.244 ** | 0.38** | 0.313** |
| Number of observations | 5960 | 5542 | 6407 | 5833 |

Multimorbidity at age 55 (NCDS) and coastal living at age 42

|  | Unadjusted | Adjusted | | Prior MM adjustment | |  |
| --- | --- | --- | --- | --- | --- | --- |
| Coastal age 42 | 1.272** | 1.159* | | 1.169* | |  |
| Neighbourhood deprivation age 42 |  | 1.091** | | 1.078** | |  |
| Female |  | 1.032 | | 1.1 | |  |
| Social class (ref: V) |  |  | |  | |  |
| VI |  | 0.674** | | 0.699** | |  |
| III manual |  | 0.783** | | 0.762** | |  |
| III non-manual |  | 0.857 | | 0.873 | |  |
| II |  | 1.203* | | 1.196* | |  |
| I |  | 0.916 | | 0.862 | |  |
| Multimorbidity age 42 |  |  | | 5.46** | |  |
| Intercept | 0.365** | 0.254** | | 0.213** | |  |
| Number of observations | 7323 | 6651 | | 6646 | |  |
|  |  |  | |  | |  |
| ** p<.01, * p<.05 | | |  | |  | |

Multimorbidity at age 46 (BCS) and coastal living at age 42

|  | Unadjusted | Adjusted | | Prior MM adjustment | |  |
| --- | --- | --- | --- | --- | --- | --- |
| Coastal age 42 | 1.136 | 1.062 | | 1.108 | |  |
| Neighbourhood deprivation age 42 |  | 1.070 ** | | 1.060 ** | |  |
| Female |  | 1.276 ** | | 1.155 * | |  |
| Social class (ref: V) |  |  | |  | |  |
| VI |  | 0.997 | | 1.005 | |  |
| III manual |  | 1.031 | | 0.985 | |  |
| III non-manual |  | 0.884 | | 0.832 | |  |
| II |  | 0.905 | | 0.868 | |  |
| I |  | 0.679 | | 0.727 | |  |
| Multimorbidity age 42 | | |  | | 8.944 ** | |
| Intercept | 0.233 ** | 0.155 ** | | 0.112 ** | |  |
| Number of observations | 7070 | 6550 | | 6538 | |  |
|  |  |  | |  | |  |
| ** p<.01, * p<.05 | | |  | |  | |

Multimorbidity at age 55 (NCDS)/ age 46 (BCS) and moving status between coastal and inland areas at age 16 and 42

|  | NCDS | BCS |
| --- | --- | --- |
| Moving status (ref: remain inland) | |  |
| Remain coastal | 0.972 | 1.049 |
| Move to coast | 1.457 ** | 1.122 |
| Move from coast | 0.868 | 0.743 |
| Neighbourhood deprivation age 16 | 1.040 ** | 1.040 ** |
| Female | 1.051 | 1.128 |
| Social class (ref: I) | |  |
| II | 0.802 | 0.892 |
| III non-manual | 0.781 * | 0.866 |
| III manual | 0.916 | 0.731 |
| IV | 1.186 | 0.81 |
| V | 0.822 | 0.709 |
| Multimorbidity age 42 | 5.603 ** | 9.128 ** |
| Intercept | 0.245 ** | 0.136 ** |
| Number of observations | 5454 | 5164 |
|  |  |  |
| ** p<.01, * p<.05 | |  |

Moving status between coastal and inland areas at age 16 and 42 and neighbourhood deprivation at age 16 and 42 (base outcome is remaining inland)

|  | NCDS | BCS |
| --- | --- | --- |
| **Remaining in coastal BUA** | |  |
| Neighbourhood deprivation age 16 | 1.074** | 1.115** |
| Neighbourhood deprivation age 42 | 1.109** | 1.097** |
|  |  |  |
| **Move to a coastal BUA** | |  |
| Neighbourhood deprivation age 16 | 0.9321** | 0.961* |
| Neighbourhood deprivation age 42 | 1.077** | 1.087** |
|  |  |  |
| **Moving from a coastal BUA** | |  |
| Neighbourhood deprivation age 16 | 1.011 | 1.061** |
| Neighbourhood deprivation age 42 | 0.9139** | 0.910** |
